# Lifestyle Changes Related to Eating Habits, Physical Activity, and Weight Status During COVID-19 Quarantine in Italy and Some European Countries

**DOI:** 10.3389/fnut.2021.718877

**Published:** 2021-08-20

**Authors:** Arianna Catucci, Umberto Scognamiglio, Laura Rossi

**Affiliations:** CREA Council for Agricultural Research and Economics – Research Centre for Food and Nutrition, Rome, Italy

**Keywords:** COVID-19, lockdown, quarantine, eating habits, physical activity

## Abstract

Novel human coronavirus disease (COVID-19), an infectious respiratory disease, has affected more than 50 million people around the world up to November 2020, thereby becoming the fifth documented pandemic since the Spanish flu in 1918. SARS-CoV-2 virus originated in China and evolved for 4 months within the country before becoming a global threat. There is currently no drug approved by the Food and Drug Administration (FDA) for which efficacy on the virus has been proved. Therefore, the only strategy against this virus is to apply measures that are capable of reducing its spread, such as isolation and quarantine, social distancing, community-wide containment, and strict enforcement of hygiene. Quarantine has proved to be effective in combating the spread of the virus; however, it has inevitably led to a radical change in the lives of people. Studies have been conducted in Italy and some European countries to highlight the role that quarantine has played in determining the lifestyle changes both in eating habits and physical activity and their possible correlation with increase in weight. The selection criteria involved answering a questionnaire that included information on the weight status and at least one of the other two aspects: changes in eating habits and/or physical activity during the quarantine period. The results obtained indicate, in general, that the negative effect of quarantine was on eating habits and physical activity. This was based on the observation that there has been an increase in food consumption and a reduction in physical activity with a consequent increase in weight.

## Introduction

Novel human coronavirus disease (COVID-19), an infectious respiratory disease, has affected more than 50 million people around the world, thereby becoming the fifth documented pandemic since the Spanish flu in 1918. COVID-19 was first reported in Wuhan city, China, where it was possible to trace the first reported case and the subsequent outbreak from a group of new cases of human pneumonia in late December 2019 (1). Human-to-human aerosol transmission is undoubtedly the main source of contagion of the virus, which occurs mainly through infected respiratory droplets, our hands, or contaminated surfaces (2). Transmission of the virus occurs when the infected respiratory droplets are ingested or inhaled by individuals in close proximity; contact transmission occurs when a person touches a surface or object contaminated with the virus and subsequently touches his/her mouth, nose, or eyes; and aerosol transmission occurs when the respiratory droplets mix with the air, forming aerosols, which may cause infection in the lungs when inhaled in high doses in a relatively closed environment (3). The median incubation period for COVID-19 is 5.1 days and it is expected that almost every person who has been infected will show symptoms within 12 days of infection (4). Symptoms of COVID-19 vary for each individual, ranging from an asymptomatic infection to severe respiratory failure (2). At present there is no drug approved by the Food and Drug Administration (FDA) for which efficacy on the virus has been proved. Therefore, the only strategy against this virus is to apply measures that are capable of reducing its spread, such as isolation and quarantine, social distancing, community-wide containment (5), and strict enforcement of hygiene. In these circumstances, governments of the countries most affected have implemented various levels of preventative measures and imposed restrictions on their citizens to contain the spread of COVID-19. Quarantine has been found to be a valuable tool in containing the spread of the virus; however, it has inevitably led to a radical change in the lives of people.

Physical distancing and self-isolation strongly impact the lives of the citizens by affecting their eating habits and everyday behavior. The two major impacts include staying at home (which includes digital education, smart working, limited outdoor activity, and in-gym physical activity) and stockpiling food due to the restrictions on grocery shopping (6). During the lockdown, boredom and stress due to the continuous streaming of news related to COVID-19 by the media can lead to overeating and the search for “comfort food” capable of reducing the stress by increasing the production of serotonin which has a positive effect on mood (7–9). In most countries where the lockdown has been enforced, movement has been restricted by allowing people to leave their homes only for essential activities; schools and universities, gymnasiums, public places, and businesses have been closed. Among the unintended consequences of quarantine, in addition to the change in eating habits, there has been a reduction in physical activity and an increase in sedentary behavior due to the excessive time spent sitting or lying down watching television or using mobile devices, and related to the closure of gyms and public parks and smart working.

Several studies have been carried out with the aim of highlighting the role of the first COVID-19 lockdown in determining lifestyle changes, both in eating habits and physical activity and their possible correlation with increase in weight.

## Materials and Methods

This study was conducted between August and November 2020. The research strategy was based on the following keywords: “coronavirus,” “COVID-19,” “lockdown,” “quarantine,” “eating habits,” and “physical activity.” PubMed, Google Scholar, and articles by research centers and public institutes related to quarantine and lifestyle changes were searched to identify studies related to the objective of the present paper.

The studies were selected based on the following eligibility criteria: (1) the study had to be conducted in Europe, and (2) the questionnaire had to include information on the weight status during lockdown and information on the lifestyle changes in eating habits and/or in the performance of physical activity during lockdown. The absence of information on the weight status in the study was an exclusion criterion. Based on these criteria, seven European studies were selected of which three were conducted in Italy, and one each was conducted in Portugal, Spain, France, and Poland.

## Results

**Figure 1:**
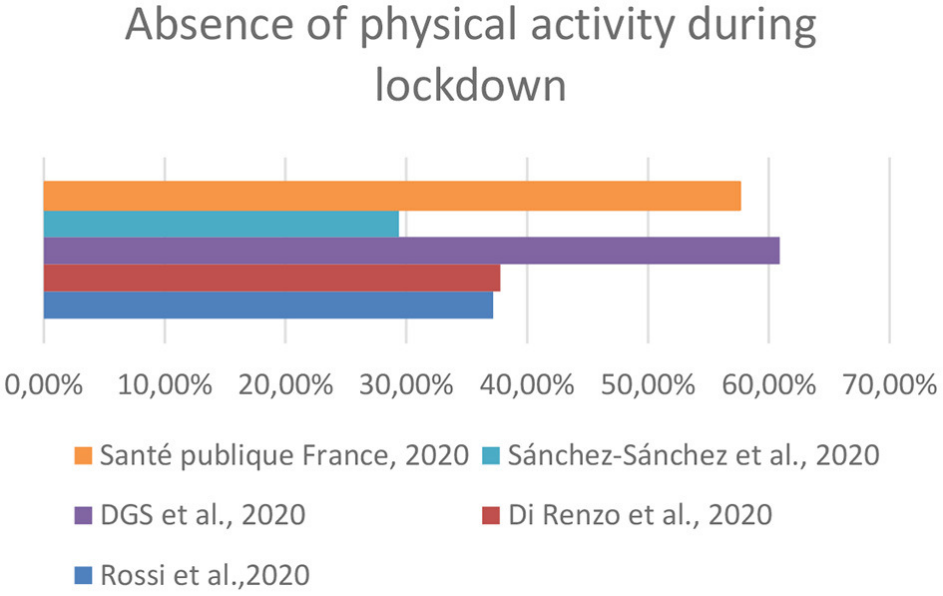
Comparison among studies regarding the absence of physical activity during lockdown.

A total of seven studies with the abovementioned inclusion criteria were selected. Each study was carried out through the dissemination of an online questionnaire, while a computer-assisted telephone interview (CATI) system was included only in the Portugal study. Participants were fully informed about the requirements of the study and were asked to fill out the questionnaire which was available online. Their personal information was anonymized to maintain and protect confidentiality. The selected studies and the characteristics of the participants are reported in Table 1. As shown in Table 1, the Italian respondents reported weight gain in different percentages based on different studies as follows: 44% based on the OERSA national survey (10), 48.6% based on EHLC-COVID19 project by the University of Rome Tor Vergata (6), and 19.5% based on the University of Padova national survey (12). The results of changes in eating habits are reported in Table 2. An increase in the intake of sweets, salty snacks, sweet beverages, and alcohol was reported. In addition, all the three studies showed an increase in the consumption of healthy foods, such as fruits and vegetables, extra virgin olive oil, and legumes. Also, hydration was better accomplished during the restriction period with a general increase in water consumption reported in the three Italian studies. In terms of intake of alcohol, it should be pointed out that even the three Italian studies confirmed a general increase in consumption, while in the EHLC-COVID19 study and the University of Padova national survey, a relevant percentage (24 and 36.8%, respectively) of respondents claimed to have reduced their consumption, probably due to a reduction in “social drinking” (data not shown). Changes in physical activity during lockdown in Italy are reported in Figure 1. The OERSA national survey and the EHLC-COVID19 project, respectively, reported a prevalence of 37.2 and 37.8% of respondents that did not perform physical activity during the lockdown. However, the answers were polarized with the two questionnaires showing a relevant quota of respondents who declared that they trained more than five times a week (17.2% for OERSA and 16.4% for EHLC-COVID19). Of particular relevance is the fact that the EHLC-COVID19 study reported an increasing percentage of people (6.1 vs. 16.4%) who performed physical activity five times a week as an effect of confinement measures (data not shown).

**Table 1:** Characteristics of the selected studies.

**Studies**	**Country**	**Questionnaire**	**Sections**	**Participants**	**Age groups (years)**	**Weight gain (%)**
OERSA national survey (10)	Italy	Online	Eating habits and physical activity	2,900	18–29 (24%) 30–49 (38.6%) 50–69 (36%)	44
EHLC-COVID19 project by University of Rome Tor Vergata (11)	Italy	Online	Eating habits and physical activity	3,533	<18 (5.1%) 18–30 (29.7%) 31–50 (42.2%) 51–65 (19.6%) >65 (3.4%)	48.6
University of Padova national survey (12)	Italy	Online	Eating habits	1,929	<20 (14.4%) 21–35 (63.1%) 36–50 (9.6%) 51– >65 (12.9%)	19.5
DGS national survey (13)	Portugal	Online and CATI	Eating habits and physical activity	5,874	≥16	26.4
University of Cádiz national survey (14)	Spain	Online	Eating habits and physical activity	1,065	16–25 (17.9%) 26–40 (39%) 41–55 (32.9%) 56–70 (9.8%) ≥71 (0.4%)	52.7
CoviPrev by Santé publique France (15)	France	Online	Eating habits and physical activity	2,010	Unspecified	27
University of Poznań national survey (16)	Poland	Online	Eating habits	1,097	18–25 (53.6%) 26–35 (28.3%) 36–45 (13.1%) >45 (4.9%)	29.9

**Table 2:** Increase in food intake during the lockdown in Italy.

**Increase in food intake during the lockdown (%)**	**OERSA national survey**	**EHLC-COVID19 project**	**University of Padova national survey**
Extra virgin olive oil	21.5	-	5
Vegetables	33	26	21.2
Fruits	29	15	
Legumes	26.5	15	-
Water	22	-	-
Dairy products	-	13	13.3
Milk and yogurt	-	9	14.3
Sweets	44.5	-	42.5
Packaging baked products	-	12	-
Homemade sweets	-	44	-
Homemade pizza	-	34	-
Fresh bread	-	18	-
Salty snacks	-	10	23.5
Sweet beverages	-	8	5
Wine	16	-	-
Alcohol (spirits, wine and beer)	-	10	10.1

In the study selected for Portugal (13), 26.4% of the respondents confirmed that they had gained weight. Again, this finding is justified by the lifestyle changes, either in terms of food intake or physical activity. With reference to eating habits, there has been an increase in the consumption of sweets, salty snacks, and alcohol. Also, in Portugal as in Italy, a relevant proportion (28.2%) of respondents claimed to have reduced their consumption. Similarities between Italy and Portugal have also been reported in terms of a positive trend in the consumption of fruits, vegetables, and water (Table 3). Sedentariness has been reported by a large proportion (60.9%) of respondents (Figure 1) and more than half of the respondents (53.6%) had decreased the level of physical activity during quarantine.

**Table 3:** Increase in food intake during the lockdown in Portugal.

**Increase in food intake during the lockdown**	**%**
Vegetables	21
Fruits	29.7
Water	31.1
Sweets	30.9
Salty snacks	8.9
Alcohol (spirits, wine and beer)	9.6

In the study selected for Spain (14), more than half of the respondents (52.7%) reported an increase in body weight with 37.3% declaring to have gained 1 to 3 kg. In term of food consumption pattern, an increase in the consumption of homemade baking products (Table 4) has been revealed. With reference to physical activity, 29.4% of the respondents declared that they did not perform any physical activity during the lockdown (Figure 1), while there was an increase in the proportion (7.9 vs. 14.5%) of those who claimed to train more than six times a week (data not shown).

**Table 4:** Increase in food intake during the lockdown in Spain.

**Increase in food intake during the lockdown**	**%**
Vegetables	0.19
Fruits	0.37
Nuts	1.12
Homemade baking	6.2
Industrial baking	1.13
Alcohol (spirits, wine and beer)	1.79

In the case of France, the results of the Santé Publique France survey were evaluated, which included sections on nutrition and physical activity during the COVID-19 emergency (15). Of the total number of respondents, 27% said that their weight had increased during the quarantine. Weight gain was mentioned most frequently by those who ate more than usual (66%) and those who had more snacks (60%). About 22% of respondents said they had increased the frequency of eating snacks. With reference to changes in physical activity and performance, 57.6% of the respondents declared that they did not perform any physical activity during the lockdown (Figure 1).

In the study selected for Poland (16), weight gain was reported by 29.9% of the respondents in line with lifestyle changes in terms of eating habits. The study examined the frequency of consumption of particular food products during quarantine (Table 5). One-fourth (25%) of the respondents did not consume vegetables and fruits on a daily basis, and the same proportion admitted to consuming sweets at least once every day. Alcohol consumption increased in 14.6% of the respondents, while 77% confirmed that they did not increase their consumption.

**Table 5:** The frequency of consumption of particular foods during quarantine in Poland.

**Frequency of consumption (%)**	**>1 per day**	**Once per day**	**Few times per week**	**Once per week**	**Once per month**	**Occasionally**	**Never**
Vegetables and fruit	25.1	42.1	25.5	5	0.7	1	0.5
Legumes	3.6	16.8	40.2	20.7	11.4	5.5	1.9
Fast food	0.3	0.7	6.7	12.8	27.8	28.3	23.4
Sweets	6.7	26.1	36.6	17.7	5.2	4.9	2.8
Salty snacks	1.5	6.3	22.6	25.3	19.8	13.7	10.8

## Discussion

Seven studies were identified in Italy, Portugal, Spain, France, and Poland, based on the preparation and dissemination of questionnaires aimed at evaluating the lifestyle changes with reference to eating habits and physical activity and which included questions concerning the weight status of the respondents, with the aim of assessing the likely correlation between the lifestyle changes caused by quarantine during COVID-19 and an increase in weight.

The results of the surveys were obtained by analyzing the responses in the self-administered questionnaires. This method of administration has several advantages such as cost reduction and a maximum degree of standardization of applications. However, the main limitation of this type of questionnaire is due to a bias among respondents resulting in a self-selection of the sample so that the parameters cannot be considered as in the application of a probabilistic sampling (17).

**Figure 2:**
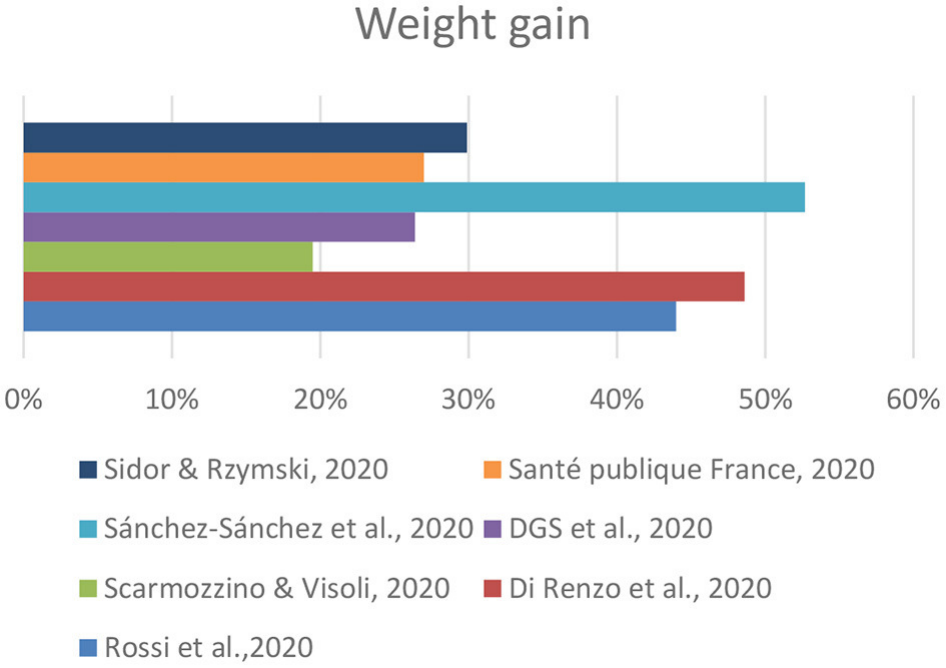
Weight gain reported in the studies.

The results indicated that in the samples of each study the increase in weight occurred in different proportions: the percentage of respondents who said they had gained weight during the lockdown ranged from 19.5% in Italy (12) to 52.7% in Spain (14) (Figure 2).

It is difficult to compare these data collected in different ways, without standardization of age groups, using different questionnaires, and affected by the sampling bias related to self-selection of respondents. Even with these limitations, the general perception of a worsening in the nutritional status with increase in weight due to the effect of quarantine could be confirmed. This weight increase is in line with the changes in eating habits and daily physical activity. In particular, there was a general tendency to eat more, with an increase in the consumption of comfort food both sweet and salty. The increase in the consumption of this type of food is probably associated with boredom and stress generated by the COVID-19 emergency, in fact, there is a clear relationship between anxiety levels and the desire for food that results in excessive consumption of calories (18). Food during this period acted as a consolatory element in an exceptional situation, which has never occurred before. The EHLC-COVID19 Project by the University of Rome Tor Vergata (11), which aimed to analyze the psychological state of the Italian population during the COVID-19 pandemic and its correlation with their eating habits in the Italian population, has highlighted that isolation, lack of stimuli, boredom, and a change in the eating habits had effects on 86% of the respondents who reported they were unable to sufficiently control their diet. Based on the study, it also emerges that there are differences based on gender regarding emotional hunger. Women display a higher state of eating anxiety compared to men, probably because the female physiology is more prone to emotional hunger and symptoms of depression (19).

Furthermore, it is interesting to observe that even in non-European countries, weight gain due to changes in the eating habits has been probably associated with the consumption of food being considered as a consolatory element. In particular, in the study conducted by Zeigler et al. (20), 52% of the participants said that they increased “eating in response to stress,” and 73% stated that they increased “eating when bored.”

However, some of the selected studies have revealed interesting data on the positive increase in the consumption of certain foods, also highlighting a tendency to eat better; in particular, the increase in the consumption of fruits and vegetables has been reported by all the three Italian studies, the Portuguese, and the Spanish studies. This aspect is noteworthy, as an increased consumption of fruits and vegetables can contribute to the enhancement of antiviral immune defense (21). In fact, an adequate intake of such foods contributes to improving the different aspects of the immune system (22), thereby providing an important antiviral prevention property/mechanism for COVID-19.

Lifestyle changes regarding physical activity also appear to be in line with the weight gain that was observed in the respondents of the different studies. Indeed, there are relevant percentages of respondents who declared the absence of or reduced physical activity in each study which included questions concerning physical activity. These results agree with those reported by the systematic review conducted by Stockweel et al., which evaluated, based on 66 studies, changes in physical activity and sedentary behavior during the COVID-19 pandemic lockdown. This review demonstrated that the majority of the studies found that physical activity declined and sedentary behavior increased during the COVID-19 lockdown. The reasons for the absence of or reduced physical activity are not clear, but this could probably be due to the closure of sports and recreational facilities and the limits imposed by the governments of different countries on the time that could be spent outdoors (23).

Interesting data emerged in relation to an increased percentage of respondents who reported having carried out physical activity more than five or more than six times a week, respectively, from the EHLC-COVI19 project by the University of Rome Tor Vergata in Italy and the national survey conducted by the University of Cádiz in Spain. These data indicate that the respondents who regularly performed physical activity before the lockdown increased their training at present probably because they had more time to train, while those who did not exercise continued not to do so.

The percentage of alcohol consumption increased during the lockdown. However, relevant data are those reported by the EHLC-COVID19 project, the University of Padova national survey, and the DGS national survey with reference to the reduction in alcohol consumption during the lockdown. This reduction is probably due to social drinking in bars and restaurants being replaced with consumption of alcohol at home.

In conclusion, the data unanimously show an effect of the quarantine on the eating habits and physical activity. The understanding of how quarantine influenced the behavior of people during the COVID-19 emergency is certainly a starting point to develop prevention and food education programs that should be put in place to correct the unhealthy habits that occur in terms of food consumption and physical activity. In reference to eating habits, in order to support individuals in eating healthy during self-quarantine and isolation, WHO has prepared a set of general tips which include limiting the consumption of salt, sugar, fat, and alcohol and encouraging the consumption of fiber, water, and fresh products, especially fruits, vegetables, and reduced-fat dairy (24).

Different countries are establishing new rules with regard to outdoor physical activity depending on the conditions related to COVID-19. Depending on the country, group or individual outdoor activities may be allowed, however, the reopening of gyms or sports centers is still uncertain. For this reason, the maintenance of an active lifestyle should also be encouraged through the performance of exercises at home or through daily activities. The WHO recommends 150 min of moderate-intensity or 75 min of vigorous-intensity physical activity for a week, or a combination of both. These recommendations can still be achieved even at home, with no special equipment and with limited space. In order to support an active lifestyle even during quarantine and isolation, the WHO has released some tips on how to stay active and reduce sedentary behavior and also prepared a set of home-based exercises (25).

## Author Contributions

The research questions and design of the study were carried out by US, AC, and LR. The methodology was revised by US. The study was carried out by AC. Data interpretation was undertaken by AC, US, and LR. Writing and original draft preparation were carried out by AC. Writing, reviewing, and editing were done by LR and US. All authors have read and agreed to the published version of the manuscript.

## Conflict of Interest

The authors declare that the research was conducted in the absence of any commercial or financial relationships that could be construed as a potential conflict of interest.

## Publisher's Note

All claims expressed in this article are solely those of the authors and do not necessarily represent those of their affiliated organizations, or those of the publisher, the editors and the reviewers. Any product that may be evaluated in this article, or claim that may be made by its manufacturer, is not guaranteed or endorsed by the publisher.

## References

[1] Liu YC Kuo RL Shih SR. COVID-19: the first documented coronavirus pandemic in history. Biomed J. (2020) 43:328–33. 10.1016/j.bj.2020.04.007 32387617 PMC7199674

[2] Pascarella G Strumia A Piliego C Bruno F Del Buono R Costa F. et al. COVID-19 diagnosis and management: a comprehensive review. J Intern Med. (2020) 288:192–206. 10.1111/joim.13091 32348588 PMC7267177

[3] Adhikari SP Meng S Wu YJ Mao YP Ye RX Wang QZ. Epidemiology, causes, clinical manifestation and diagnosis, prevention and control of coronavirus disease (COVID-19) during the early outbreak period: a scoping review. Infect Dis Poverty. (2020) 9:29. 10.1186/s40249-020-00646-x 32183901 PMC7079521

[4] Lauer SA Grantz KH Bi Q Jones FK Zheng Q Meredith HR. The incubation period of coronavirus disease 2019 (COVID-19) from publicly reported confirmed cases: estimation and application. Ann Intern Med. (2020) 172:577–82. 10.7326/M20-0504 32150748 PMC7081172

[5] Wilder-Smith A Freedman DO. Isolation, quarantine, social distancing and community containment: pivotal role for old-style public health measures in the novel coronavirus (2019-nCoV) outbreak. J Travel Med. (2020) 27:taaa020. 10.1093/jtm/taaa020 32052841 PMC7107565

[6] Di Renzo L Gualtieri P Pivari F Soldati L Attinà A Cinelli G. Eating habits and lifestyle changes during COVID-19 lockdown: an Italian survey. J Transl Med. (2020) 18:229. 10.1186/s12967-020-02399-5 32513197 PMC7278251

[7] Moynihan AB van Tilburg WA Igou ER Wisman A Donnelly AE Mulcaire JB. Eaten up by boredom: consuming food to escape awareness of the bored self. Front Psychol. (2015) 6:369. 10.3389/fpsyg.2015.00369 25883579 PMC4381486

[8] Rodríguez-Martín BC Meule A. Food craving: new contributions on its assessment, moderators, and consequences. Front Psychol. (2015) 6:21. 10.3389/fpsyg.2015.00021 25657636 PMC4302707

[9] Ma Y Ratnasabapathy R Gardiner J. Carbohydrate craving: not everything is sweet. Curr Opin Clin Nutr Metab Care. (2017) 20:261–5. 10.1097/MCO.0000000000000374 28375878 PMC5837018

[10] OERSA. Available online at: https://www.crea.gov.it/-/covid-19-come-sono-cambiate-le-nostre-abitudini-alimentari-durante-il-lockdown-?inheritRedirect=true&redirect=%2Fricerca%3Fq%3Dcome%2520sono%2520cambiate%2520le%2520nostre%2520abitudini (accessed 21 May, 2021).

[11] Di Renzo L Gualtieri P Cinelli G Bigioni G Soldati L Attinà A. Psychological aspects and eating habits during COVID-19 home confinement: results of EHLC-COVID-19 Italian online survey. Nutrients. (2020) 12:2152. 10.3390/nu12072152 32707724 PMC7401000

[12] Scarmozzino F Visioli F. Covid-19 and the subsequent lockdown modified dietary habits of almost half the population in an Italian sample. Foods. (2020) 9:675. 10.3390/foods9050675 32466106 PMC7278864

[13] Direção-Geral da Saúde (DGS) Instituto de Saúde Ambiental da Faculdade de Medicina da Universidade de Lisboa. Available online at: https://www.dgs.pt/programa-nacional-para-a-promocao-da-atvidade-fisica/ficheiros-externos-pnpaf/rel_resultados-survey-covid-19-pdf.aspx (accessed 21 May, 2021).

[14] Sánchez-Sánchez E Ramírez-Vargas G Avellaneda-López Y Orellana-Pecino JI García-Marín E Díaz-Jimenez J. Eating habits and physical activity of the spanish population during the COVID-19 pandemic period. Nutrients. (2020) 12:2826. 10.3390/nu12092826 32942695 PMC7551353

[15] Santé publique France. Available online at: https://www.santepubliquefrance.fr/etudes-et-enquetes/covid-19-une-enquete-pour-suivre-l-evolution-des-comportements-et-de-la-sante-mentale-pendant-l-epidemie (accessed 21 May, 2021).

[16] Sidor A Rzymski P. Dietary choices and habits during COVID-19 lockdown: experience from Poland. Nutrients. (2020) 12:1657. 10.3390/nu12061657 32503173 PMC7352682

[17] Department for Legal and Legislative Affairs (Presidency of the Council of Ministers) 2013. Available online at: http://www.qualitanormazione.gov.it/uploads/download/file/270/Strumenti_per_il_ciclo_della_regolazione.pdf (accessed 21 May, 2021).

[18] Recio-Román A Recio-Menéndez M Román-González MV. Food reward and food choice. An inquiry through the liking and wanting model. Nutrients. (2020) 12:639. 10.3390/nu12030639 32121145 PMC7146242

[19] Iddir M Brito A Dingeo G Del Campo SSF Samouda H La Frano MR. Strengthening the immune system and reducing inflammation and oxidative stress through diet and nutrition: considerations during the COVID-19 crisis. Nutrients. (2020) 12:1562. 10.3390/nu12061562 32471251 PMC7352291

[20] Zeigler Z Forbes B Lopez B Pedersen G Welty J Deyo A. Self-quarantine and weight gain related risk factors during the COVID-19 pandemic. Obes Res Clin Pract. (2020) 14:210–6. 10.1007/s13679-021-00449-7 32460966 PMC7241331

[21] Alkhatib A. Antiviral functional foods and exercise lifestyle prevention of coronavirus. Nutrients. (2020) 12:2633. 10.3390/nu12092633 32872374 PMC7551447

[22] Gombart AF Pierre A Maggini S. A review of micronutrients and the immune system–working in harmony to reduce the risk of infection. Nutrients. (2020) 12:236. 10.3390/nu12010236 31963293 PMC7019735

[23] Stockwell S Trott M Tully M Shin J Barnett Y Butler L. Changes in physical activity and sedentary behaviours from before to during the COVID-19 pandemic lockdown: a systematic review. BMJ Open Sport Exerc Med. (2021) 7:e000960. 10.1136/bmjsem-2020-000960 34192010 PMC7852071

[24] WHO Food and nutrition tips during self-quarantine. Available online at: https://www.euro.who.int/en/health-topics/health-emergencies/coronavirus-covid-19/publications-and-technical-guidance/food-and-nutrition-tips-during-self-quarantine (accessed on 21 May 2021a).

[25] WHO Stay physically active during self-quarantine. Available online at: https://www.euro.who.int/en/health-topics/health-emergencies/coronavirus-covid-19/publications-and-technical-guidance/noncommunicable-diseases/stay-physically-active-during-self-quarantine (accessed on 21 May 2021b).

